# Endoplasmic Reticulum Stress Mediates Axon Initial Segment Shortening: Implications for Diabetic Brain Complications

**DOI:** 10.1007/s12031-025-02448-y

**Published:** 2025-12-19

**Authors:** Jennae N. Shelby, Amanda M. Chisholm, Islam Akhmedov, Nathan Sheriff, Ryan B. Griggs, Keiichiro Susuki

**Affiliations:** Department of Neuroscience, Cell Biology, and Physiology, Boonshoft School of Medicine, Wright State University, 3640 Colonel Glenn Highway, Dayton, OH 45435 USA

**Keywords:** Axon initial segment, Endoplasmic reticulum stress, Protein kinase RNA-like ER kinase, Methylglyoxal, Type 2 diabetes mellitus

## Abstract

Endoplasmic reticulum (ER) stress and activation of the three unfolded protein response pathways, in particular the protein kinase RNA-like ER kinase (PERK) pathway, contribute to the pathophysiology of various neurodegenerative conditions including type 2 diabetes mellitus (T2DM). T2DM is an increasingly prevalent metabolic disorder affecting millions. Even with strict glucose control, patients with T2DM frequently experience mild cognitive impairment and exhibit a significantly increased risk of developing dementia. We previously demonstrated that impaired cognitive flexibility is associated with shortening of axon initial segment (AIS) length in the prefrontal cortex in the T2DM model *db/db* mice. The AIS plays the crucial roles of regulation of action potential initiation and neuronal output. Even subtle shortening of AIS length can reduce excitability of neurons. In this study, we hypothesized that ER stress mediates AIS shortening in diabetic conditions. Utilizing primary mouse cortical cultures, we show that sodium 4-phenylbutyrate, a well-documented ER stress inhibitor, prevents AIS shortening and PERK activation induced by the T2DM factor methylglyoxal. Exposure of cortical cultures to an established ER stress inducer tunicamycin caused dose-dependent reduction of AIS length in the generalized population of the neurons without affecting neuronal viability. Co-exposure to a PERK-specific inhibitor GSK2606414 prevented AIS shortening induced by tunicamycin. These results demonstrate ER stress is sufficient and necessary for AIS shortening in vitro. Our findings identify ER stress and AIS shortening as potential therapeutic targets in T2DM-related cognitive impairment.

**Supplementary Information:**

The online version contains supplementary material available at 10.1007/s12031-025-02448-y.

## Introduction

Type 2 diabetes mellitus (T2DM) is a condition characterized by impaired glucose metabolism that affects millions of patients and is increasing in prevalence globally at an alarming rate (Khan et al. 2020). Patients with diabetes are 1.6 times more likely to develop dementia, and mild cognitive impairment is frequently experienced in those without dementia (Biessels and Despa 2018; Livingston et al. 2020). The prevalence of mild cognitive impairment in T2DM patients was estimated to be 45.0% (You et al. 2021). Impaired cognitive function can lead to poor self-management of diabetes (e.g. blood glucose monitoring), resulting in more frequent diabetic complications and hospital admissions (Srikanth et al. 2020). However, few recommendations are currently available for how to modify the course of cognitive impairment (Srikanth et al. 2020). Importantly, a Cochrane review found no strong evidence that any established treatment strategies for T2DM can prevent or delay cognitive impairment (Areosa Sastre et al. 2017), indicating that specific treatments targeting cognitive functions are required. Despite being a well-described comorbidity, the specific mechanisms underlying the cognitive impairment that occurs in T2DM remain unknown. These existing gaps in knowledge and adverse outcomes present a critical need to identify the mediators facilitating the cognitive impairment found in T2DM patients.

The axon initial segment (AIS) located next to the soma is crucial for initiation of action potentials and regulation of neuronal outputs (Rasband 2010; Bender and Trussell 2012; Leterrier 2018; Fréal and Hoogenraad 2025). The AIS is characterized by highly accumulated ion channels such as voltage-gated sodium channels, a scaffolding protein AnkyrinG, and a sub-membranous cytoskeletal protein βIV spectrin. Action potentials are initiated at the AIS by voltage-gated sodium channels. Emerging evidence indicates that the altered AIS structures play key roles in the pathophysiology of various neurological diseases (Buffington and Rasband 2011; Huang and Rasband 2018; Garrido 2023; Fréal and Hoogenraad 2025). The AIS geometry such as length and start location is closely associated with neuronal excitability (Jamann et al. 2018). For example, even subtle shortening of the AIS length decreases neuronal excitability (Baalman et al. 2013; Evans et al. 2015; Jamann et al. 2021). Importantly, AIS shortening is found in an established model for T2DM, *db/db* mice, with cognitive impairment (Yermakov et al. 2018, 2019). We have previously demonstrated that increase of methylglyoxal, a highly reactive byproduct of the metabolism of glucose, induces AIS shortening in mouse primary cortical neuron culture (Griggs et al. 2021). Methylglyoxal is elevated in T2DM patients and in *db/db* mice and is implicated in diabetic complications such as painful diabetic neuropathy (Bierhaus et al. 2012; Griggs et al. 2019). Detailed mechanisms of how methylglyoxal induces AIS shortening remains to be defined.

The endoplasmic reticulum (ER) plays many roles that are vital to cellular homeostasis including maintenance of intracellular calcium levels and the modification, folding, and targeting of proteins (Walter and Ron 2011; Ghemrawi and Khair 2020). The term “ER stress” refers to a state of mismatch between the ER protein load and its capacity to fold and excrete proteins. The accumulation of unfolded proteins induces ER stress and subsequent activation of the adaptive unfolded protein response (UPR) for restoration of ER’s function. The UPR is activated by three sensor proteins: protein kinase RNA-like ER kinase (PERK), inositol-requiring transmembrane kinase/endoribonuclease 1α, and activating transcription factor 6 (Ghemrawi and Khair 2020). The ER stress and UPR have been shown to be involved in the pathogenesis of many neurodegenerative conditions. For example, ER stress and PERK activation occur in the brain of *db/db* mice with cognitive impairment (Li et al. 2018; Ye et al. 2018; Hu et al. 2020; Wu et al. 2020). Importantly, increased levels of methylglyoxal increase several key markers of UPR activation including PERK pathway (Palsamy et al. 2014; Barragán-Iglesias et al. 2019; Irshad et al. 2019; Xue et al. 2024). A structure termed the cisternal organelle formed by ER may play important roles for AIS plasticity during development (Schlüter et al. 2017). Therefore, we reasoned that ER stress may mediate AIS shortening in diabetic conditions. To test this idea, we pharmacologically modulated ER stress in mouse cortical neuron culture and evaluated AIS geometry.

## Materials and Methods

### Primary Mouse Cortical Culture

Mouse cortical culture was prepared as described before (Griggs et al. 2021). C57BL/6J mice (The Jackson Laboratory) were housed at Wright State University and used to collect neonate brains. Mice were housed at 22–24 °C temperature, 20–60% humidity, and 12-hour light/12-hour dark cycle, with food and water ad libitum. Postnatal day (P) 0 mouse pups (male and female) were euthanized by decapitation, cortices were removed, and cells were dissociated. All animal procedures were performed in compliance with the National Institutes of Health Office of Laboratory Animal Welfare Guide for the Care and Use of Laboratory Animals and the ARRIVE guidelines. The experimental protocol was approved by the Wright State University Institutional Animal Care and Use Committee (#1190). In some experiments, embryonic day (E)18 mouse dissociated cortices (SKU C57EDCX) were purchased from Transnetyx Tissue. Cells were plated in 35 mm dishes coated with poly-L-lysine for protein analyses or on coverslips coated with poly-L-lysine for immunofluorescence, and were maintained in standard growth media (Neurobasal Plus, 2 mM (final) GlutaMAX, 2% (final) B-27 Plus) in a humidified atmosphere at 37 °C with 5% CO_2_.

### Drug Exposure

Because the ER may affect AIS length maturation (Schlüter et al. 2017), experiments were started after 10 days in vitro (DIV) to evaluate the effects of drug treatment on fully formed AISs. Methylglyoxal (M0252; Sigma-Aldrich), sodium 4-phenylbutyrate (4PBA) (SML0309; Sigma-Aldrich), tunicamycin (T7765; Sigma-Aldrich), or GSK2606414 (HY-18072; MedChemExpress) were diluted in standard growth media (2x final concentration), sterilized by the syringe-filter, and pre-warmed to 37 °C. Then, 1 mL of experimental media and 1 mL of conditioned media were added to 35 mm dishes for a 1x final concentration.

### Western Blotting of Cortical Cultures

Cell lysates for immunoblotting were prepared as we have done before (Griggs et al. 2021). In brief, after rinsing culture dishes in ice-cold PBS, cells were collected in ice-cold lysis buffer, vortexed, incubated on ice for 10 min, centrifuged, then supernatant was collected. A BCA assay (23235, Thermo Fisher Scientific) was used to measure protein concentrations. Samples were heated at 95 °C for 5 min, 10 µg of each lysate were run on 4–12% gels, then transferred to nitrocellulose membrane. The membrane used for MG-H1 immunoblotting (Griggs et al. 2021) was stripped with Restore™ Western Blot Stripping Buffer (21059, Thermo Fisher Scientific), then washed. After blocking for 1 h in 20 mM Tris, pH 8.0 and 0.05% (v/v) Tween 20 (TBST) containing 4% (w/v) milk, the membrane was incubated overnight at 4 °C in antibody to glucose regulated protein 78 (GRP78) (ADI-SPA-826, Enzo Life Sciences, RRID: AB_10617248), then washed 3 times in TBST. Horseradish peroxidase conjugated secondary antibody (Jackson ImmunoResearch Laboratories) was incubated at room temperature for 1 h, then the membrane was washed 3 times in TBST. Signals produced by a Pierce™ ECL Plus Western Blotting Substrate (32132, Thermo Fisher Scientific) were imaged using Azure 600 imaging system (Azure Biosystems). ImageLab software (Bio-Rad) was used for quantification of the band densities. The GRP78 band densities were normalized to the total protein staining by SYPRO Ruby (S4942, Sigma-Aldrich).

### Immunofluorescent Analysis of Cortical Cultures

Cortical cultures were immunostained as we have done before (Griggs et al. 2021). In brief, after washing in warm PBS, cells were fixed with 4% paraformaldehyde at room temperature for 20 min and washed 3 times in PBS. After blocking in PBSTGS (10% goat serum, 0.3% Triton X-100, PBS) at room temperature for 1 h, coverslips were incubated in primary antibody at 4 °C overnight and washed 3 times in PBSTGS. To label the AIS, AnkyrinG (N106/36, 75–146, Antibodies Incorporated, RRID: AB_10673030) or βIV spectrin (M.N. Rasband, Baylor College of Medicine, Texas, USA, RRID: AB_2315634) antibodies were used. To label neuronal soma and dendrites, antibody to microtubule associated protein 2 (MAP2) (CPCA-MAP2, EnCor Biotechnology, RRID: AB_2138173) was used. Then the coverslips were incubated with fluorescent secondary antibodies (Alexa Fluor 594, Alexa Fluor 488, or AMCA) (Jackson ImmunoResearch Laboratories) at room temperature for 1 h, washed in PBSTGS, 0.01 M PB, and 0.005 M PB. Hoechst 33258 (Thermo Fisher Scientific) was used to identify nuclei in some samples.

### AIS Measurement

To quantify AIS length, images were taken of neuron cultures with an Axio Observer Z1 microscope (Carl Zeiss), and ZEN Blue 3.0 was used to export images for use in ImageJ. A custom script was used to isolate each AIS and remove background immunofluorescence for easier processing in MATLAB. We amended MATLAB files written by Matthew Grubb (MATLAB Central File Exchange; https://www.mathworks.com/matlabcentral/fileexchange/28181-ais-quantification) to automate plotting of each AIS and measurement of the length that falls within 33% of the maximum intensity of the plotted segment. To quantify AIS start location, the soma boundary was determined by overlaying the MAP2 staining with a smooth curve along the edge of the neuron as previously done (Guo et al. 2017; Griggs et al. 2021). The distance between the soma boundary and the start position of the AIS was measured using ZEN Blue 3.0. AIS measurements for all experiments were completed under observer blinded conditions.

### P-PERK/PERK Measurement

Phosphorylated PERK (P-PERK)/PERK ratio was measured in the same cultures where the AIS lengths were measured. After the imaging of AIS structures, the primary and secondary antibodies were stripped from the coverslips using a stripping buffer (Micheva et al. 2010) composed of 0.02% sodium dodecyl sulfate and 0.2 M NaOH solution prepared with Milli-Q water. After soaking the slides in PBS overnight at 4 °C, previously mounted coverslips were gently removed and were washed 3 times for 15 min to remove all remaining mounting medium. Coverslips were then soaked in stripping buffer at room temperature for 30 min and were washed 3 times for 15 min in PBS. After verifying complete removal of immunostaining, coverslips were immunostained as described above. Antibodies targeting phosphorylated PERK (P-PERK) (Thr982) (PA5-102853, Thermo Fisher Scientific, RRID: AB_2815938) and PERK (bsm-51385M, Bioss Antibodies) were used to evaluate ER stress. 40x images were taken of the immunostained coverslips with an Axio Observer Z1 microscope, utilizing the same exposure time for each image. The intensity sums of the P-PERK (Alexa Fluor 488) and PERK (Alexa Fluor 594) fluorescence channels for each field of view were calculated with ZEN software. The intensity sum of P-PERK was divided by the intensity sum of PERK for each image (3 culture preparations, 26–36 images per culture preparation). For each preparation, each P-PERK/PERK ratio was divided by the mean of all the P-PERK/PERK ratios in the dataset. This transformation can reduce variation between preparations, while preserving the magnitude of change observed between groups.

### Cell Viability Assay

The viability of mature cortical cultures exposed to vehicle or tunicamycin was quantified based on immunofluorescence staining using a LIVE/DEAD™ Viability/Cytotoxicity Assay Kit (L3224, Thermo Fisher Scientific) following instruction provided by the manufacturer. After gently washing culture dishes 3 times in PBS, cells were incubated with LIVE/DEAD™ kit dyes at room temperature for 20 min. Three 0.6 mm^2^ areas were analyzed for each coverslip, and three coverslips were analyzed from each group per *n*. The percentage of number of live cells divided by the total cell number was reported as the cell viability. This number was normalized to the mean cell viability of the vehicle control groups.

### Statistics

One-way ANOVA with post-hoc Dunnett’s multiple comparison test was used to analyze methylglyoxal dose responses of GRP78 levels, Live/Dead assay after tunicamycin exposure, and tunicamycin dose responses of AIS length. Two-way ANOVA with post-hoc Tukey test was used to compare AIS length and P-PERK/PERK ratio in vehicle, methylglyoxal, 4PBA, and methylglyoxal + 4PBA, and AIS length in vehicle, tunicamycin, GSK2606414, and tunicamycin + GSK2606414. Pearson r correlation was used to evaluate the relationship between AIS length and P-PERK/PERK ratio. Unpaired t-test was used to compare AIS length or location between vehicle and tunicamycin exposed groups. Statistical significance was defined by an α value of 0.05. Prism 10.5.0 (GraphPad) was used for all statistical analyses and generation of graphs.

## Results

### Methylglyoxal-Induced AIS Shortening is Associated with ER Stress

**Fig. 1:**
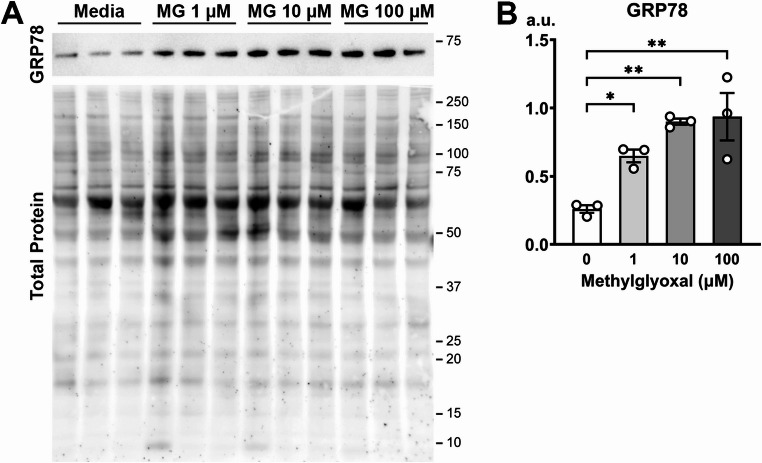
Methylglyoxal increases the ER stress chaperone GRP78. (**A**) Images of immunoblot probed for GRP78 and fluorescent stain for total protein (SYPRO Ruby). P0 mouse cortical cultures were exposed to methylglyoxal (MG; 0, 1, 10, 100 µM for 24 h, DIV 13–14). An uncropped image of the entire immunoblot is available in Online Resource Supplementary Figure S1. (**B**) Western immunoblotting results showing GRP78 protein levels. n = 3 culture preparations; One-way ANOVA with post-hoc Dunnett’s multiple comparisons test. a.u. (arbitrary units). *p < 0.05, **p < 0.01

ER stress occurs in the brains of the T2DM model *db/db* mice (Sims-Robinson et al. 2012; Li et al. 2018, 2020; Ye et al. 2018; Hu et al. 2020; Wu et al. 2020). In addition, high glucose concentration and increase of methylglyoxal can induce ER stress and subsequent UPR (Palsamy et al. 2014; Barragán-Iglesias et al. 2019; Irshad et al. 2019). For example, 1 µM methylglyoxal increased the protein level of an ER stress marker GRP78 (also known as BiP) in cultured dorsal root ganglion neurons (Barragán-Iglesias et al. 2019). To further confirm these observations, we exposed mouse cortical cultures to methylglyoxal (0, 1, 10, 100 µM) for 24 h and analyzed the expression of GRP78. Western blot of cortical culture cell lysates showed that the GRP78 expression was increased by 1 µM (*p* = 0.0418), 10 µM (*p* = 0.0030), and 100 µM (*p* = 0.0021) methylglyoxal compared to media control (One-way ANOVA (*F* (3,8) = 11.56; *p* = 0.0028) with post-hoc Dunnett’s multiple comparison test, *n* = 3 culture preparations) (Fig. 1). Similar to the AIS shortening in *db/db* mice (Yermakov et al. 2018), methylglyoxal (100 µM for 24 h) induces AIS shortening in primary mouse cortical cultures (Griggs et al. 2021). Therefore, these data demonstrate that ER stress is associated with AIS shortening in diabetic conditions.

### ER Stress Inhibition Prevents Methylglyoxal-Induced AIS Shortening

**Fig. 2:**
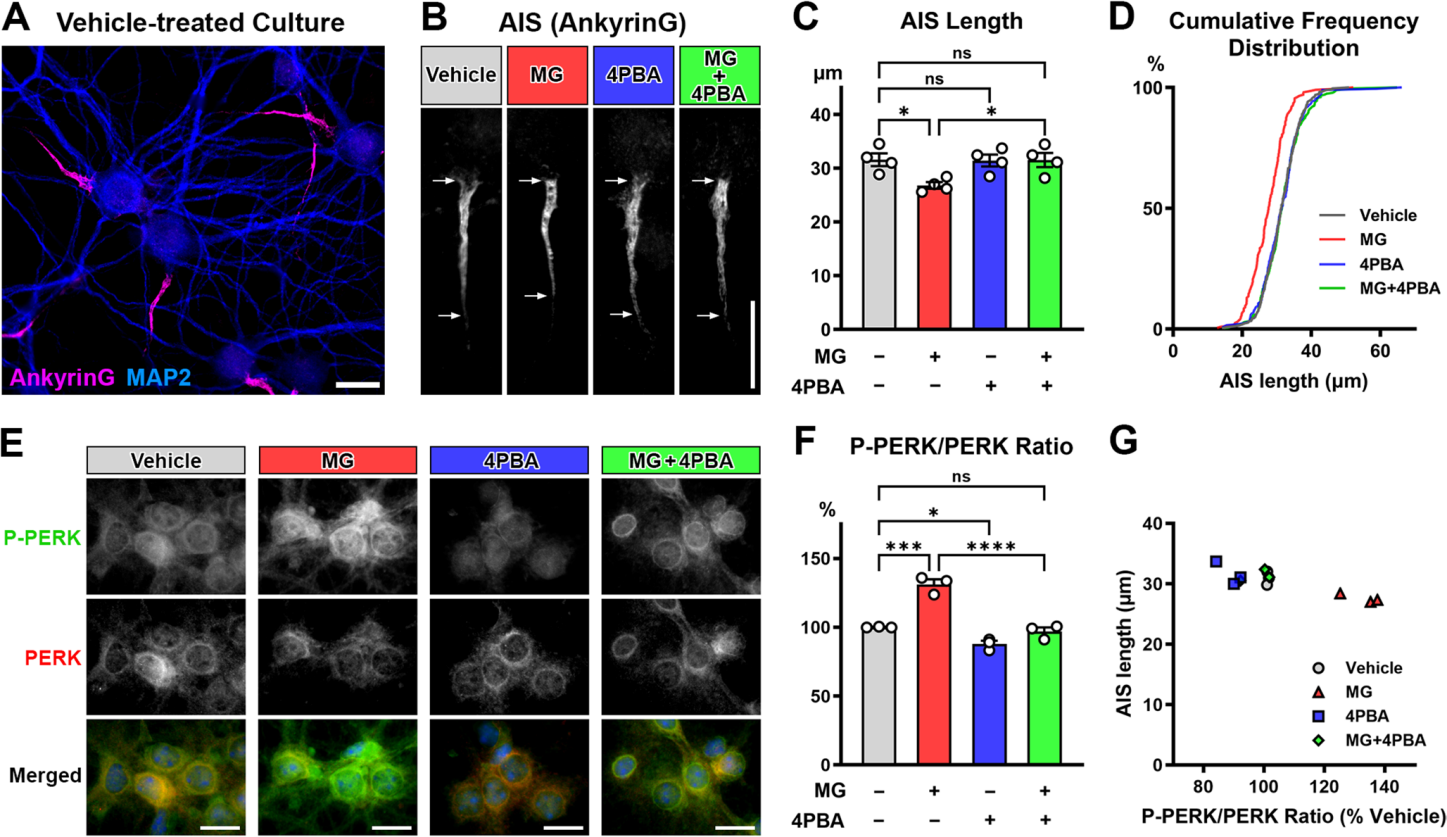
ER Stress inhibition prevents AIS shortening by the T2DM factor, methylglyoxal. (**A**) Representative image of primary mouse P0 cortical culture at DIV11 (control preparation exposed to vehicle) showing AnkyrinG (violet) for AIS and MAP2 (blue) for somatodendritic domain. Scale bar = 20 μm. (**B**) Representative images of AnkyrinG immunostaining. Arrows indicate start and end positions of the AIS. Cultures were exposed to vehicle, methylglyoxal (MG) (100 µM), an ER stress inhibitor 4PBA (100 µM), or MG + 4PBA for 24 h at DIV 11. Scale bar = 20 μm. (**C**) AIS length in mouse cortical neurons exposed to vehicle, MG (100 µM), 4PBA (100 µM), or MG and 4PBA for 24 h. Length of AIS were measured by AnkyrinG immunostaining, and average AIS length was calculated for each culture preparation (68–279 AISs per culture). Two-way ANOVA with post-hoc Tukey’s multiple comparison test (*n* = 4 culture preparations): vehicle vs. MG (*p* = 0.0390), vehicle vs. 4PBA (*p* = 0.9994), vehicle vs. MG + 4PBA (*p* > 0.9999), MG vs. 4PBA (*p* = 0.0474), MG vs. MG + 4PBA (*p* = 0.0417), and 4PBA vs. MG + 4PBA (*p* = 0.9998). *p < 0.05. (**D**) Cumulative frequency distribution of the length of all AIS (621 AISs in vehicle, 513 AISs in MG, 520 AISs in 4PBA, 654 AISs in MG + 4PBA). A leftward shift of AIS length in the MG group suggests generalized AIS shortening across the population of neurons. (**E**) Representative images of primary mouse P0 cortical culture immunostained for P-PERK (green), PERK (red), and Hoechst staining (blue). Scale bars = 20 μm. (**F**) The ratio of P-PERK to PERK measured by immunofluorescence intensity in mouse cortical cultures exposed to vehicle, methylglyoxal (MG, 100 µM), 4PBA (100 µM), or MG and 4PBA for 24 h. Two-way ANOVA with post-hoc Tukey’s multiple comparison test (*n* = 3 culture preparations): vehicle vs. MG (*p* = 0.0002), vehicle vs. 4PBA (*p* = 0.0461), vehicle vs. MG + 4PBA (*p* = 0.8389), MG vs. 4PBA (*p* < 0.0001), MG vs. MG + 4PBA (*p* < 0.0001), and 4PBA vs. MG + 4PBA (*p* = 0.1482). *p < 0.05, ***p < 0.001, ****p < 0.0001. (**G**) Scatter plot of AIS length in relation to P-PERK/PERK ratio

Next, we investigated the role of ER stress in methylglyoxal-evoked AIS shortening utilizing 4PBA, a well-documented inhibitor of ER stress. 4PBA acts as a chemical protein chaperone to reduce the protein load of the ER (Yam et al. 2007; Wang et al. 2016; Kong et al. 2018). Primary mouse cortical cultures were exposed to either media vehicle, methylglyoxal (100 µM), 4PBA (100 µM), or methylglyoxal and 4PBA for 24 h, then AIS morphology was examined by immunofluorescence (Fig. 2A, B). There was a statistically significant interaction between the effects of MG and 4PBA on AIS length (two-way ANOVA; *F* (1,12) = 5.080; *p* = 0.0437; post-hoc Tukey’s multiple comparison test; *n* = 4 culture preparations) (Fig. 2C). The AISs of cultures exposed to methylglyoxal (26.80 ± 0.6127 μm, *n* = 4 culture preparations) were significantly shorter (*p* = 0.0390) than vehicle-treated controls (31.60 ± 1.182 μm, *n* = 4 culture preparations) after 24 h (Fig. 2C, D), as previously published (Griggs et al. 2021). The AISs in methylglyoxal-treated cultures were also shorter than 4PBA and methylglyoxal co-treated cultures (31.54 ± 1.318 μm, *n* = 4 culture preparations; *p* = 0.0417). There were no differences in AIS length between vehicle controls and 4PBA treated cultures (31.42 ± 1.116 μm, *n* = 4 culture preparations; *p* = 0.9994) or 4PBA and methylglyoxal co-treated cultures (*p* > 0.9999). These data indicate that the 4PBA treatment prevents AIS shortening induced by methylglyoxal.

Previous studies have demonstrated that methylglyoxal induces ER stress and activates the PERK pathway in UPR (Palsamy et al. 2014; Barragán-Iglesias et al. 2019; Xue et al. 2024). To test if AIS changes are associated with ER stress induction and PERK activation, the primary and secondary antibodies were stripped after the imaging of AIS structures, and these cultures were re-probed for activated (phosphorylated) and inactivated forms of PERK (Fig. 2E). Upon activation, PERK is autophosphorylated (Rozpędek-Kamińska et al. 2020). The ratio of phosphorylated to unphosphorylated form of PERK (P-PERK/PERK ratio) is used as a marker of ER stress and UPR activation (Li et al. 2018; Ye et al. 2018; Wu et al. 2020). There was a statistically significant interaction between the effects of MG and 4PBA on P-PERK/PERK ratio (two-way ANOVA; *F* (1,8) = 17.03; *p* = 0.0033; post-hoc Tukey’s multiple comparison test; *n* = 3 culture preparations) (Fig. 2F). Cortical cultures treated with methylglyoxal (100 µM for 24 h) showed 131% (± 3.737, *n* = 3 culture preparations; *p* = 0.0002) the P-PERK/PERK ratio when compared to the average P-PERK/PERK ratio of vehicle controls (100%±0.1879, *n* = 3 culture preparations) (Fig. 2F). This result is consistent with an increase of GRP78 after methylglyoxal exposure (Fig. 1), indicating methylglyoxal induced ER stress. As expected, 4PBA prevented the increase in P-PERK/PERK ratio caused by methylglyoxal (97%±2.927, *n* = 3 culture preparations; *p* = 0.8389) (Fig. 2F). 4PBA and vehicle co-treated cultures had 88% (± 2.378, *n* = 3 culture preparations; *p* = 0.0461) of the vehicle P-PERK/PERK ratio. These results indicate that 4PBA effectively inhibits ER stress and AIS shortening induced by methylglyoxal in the mouse cortical culture. Finally, we observed an inverse correlation (Pearson *r* = −0.8552; 95% CI = −0.9586 to −0.5526; *p* = 0.0004) between AIS length and P-PERK/PERK ratio (Fig. 2G). These results suggest that ER stress is a key mechanism mediating AIS shortening in diabetic conditions.

### Low Dose Tunicamycin Does Not Induce Cell Death in Mouse Cortical Cultures

**Fig. 3:**
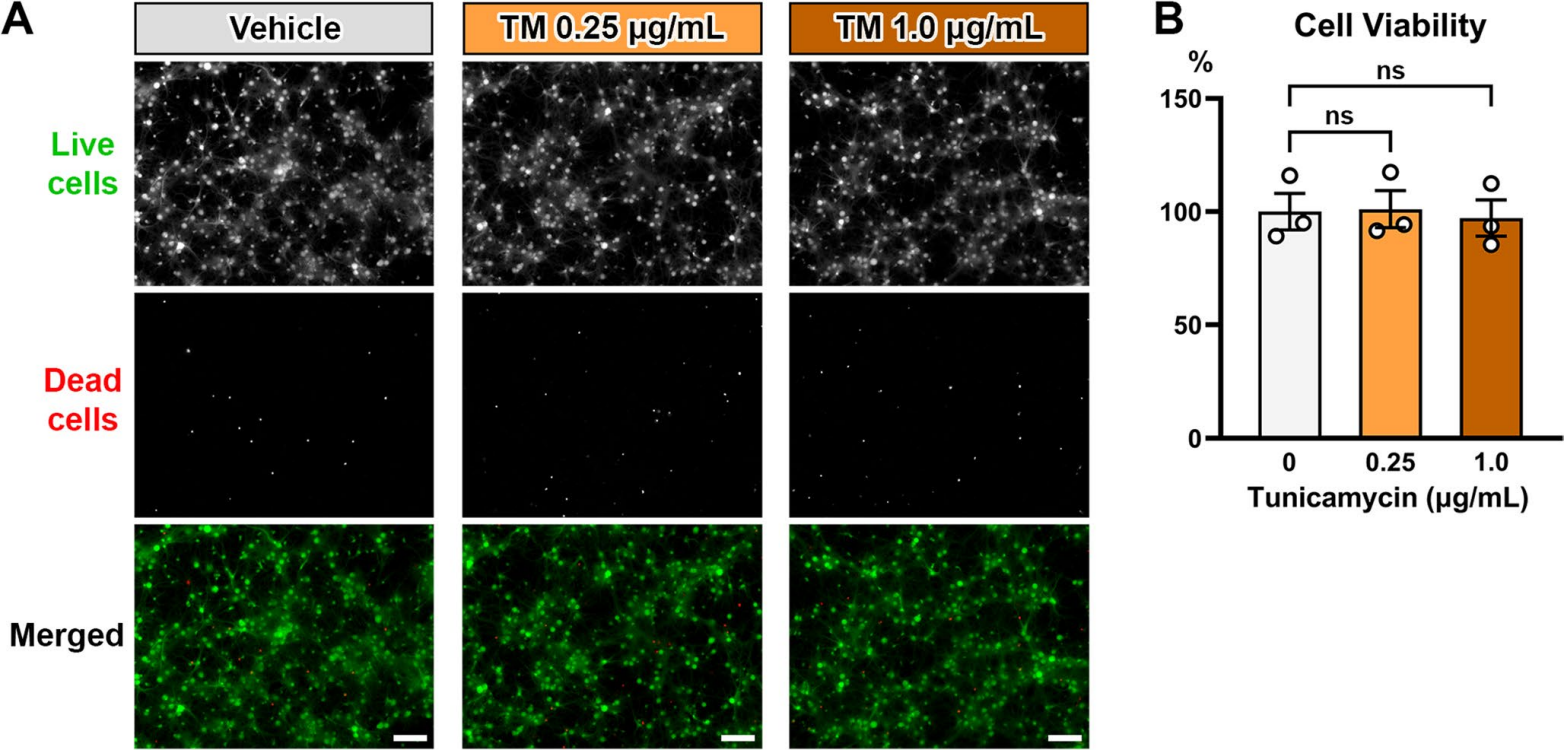
Low-dose tunicamycin does not induce cell death. (**A**) Representative images of cell viability assay showing live cells (green) and dead cells (red). E18 mouse cortical cultures were exposed to vehicle (0.025% DMSO) or tunicamycin (TM) (0.25–1.0 µg/mL) for 24 h. Scale bars = 100 μm. (**B**) The graph showing quantification of cell viability. The percentage of live cells were normalized to vehicle control. Each *n* represents the average of three coverslips from each treatment group in one culture preparation. One-way ANOVA with post-hoc Dunnett’s multiple comparisons test (*n* = 3 culture preparations): vehicle vs. 0.25 µg/mL tunicamycin (*p* = 0.9931), vehicle vs. 1.0 µg/mL tunicamycin (*p* = 0.9562)

Next, we sought to examine the impact of ER stress induction, independent of methylglyoxal or diabetic conditions, on AIS morphology in vitro. We used an established ER stress inducer tunicamycin which causes accumulation of the unfolded glycoproteins in the ER by inhibiting the synthesis of glycoproteins (Oslowski and Urano 2011). Although the apoptosis mediated by ER stress has been well studied, we are focused on the mechanisms underlying AIS shortening in the neurons without cell death (Yermakov et al. 2018; Griggs et al. 2021). We used a maximum tunicamycin dose of 1 µg/mL, because 24-hour exposure to 1 µg/mL of tunicamycin does not alter dendritic spine density or expression of synaptic proteins in primary neuron cultures (Shih and Hsueh 2016). We confirmed that 0.25–1.0 µg/mL of tunicamycin did not significantly affect cellular viability compared with vehicle control (One-way ANOVA (*F* (2,6) = 0.06364; *p* = 0.9390; post-hoc Dunnett’s multiple comparison test; *n* = 3 culture preparations) (Fig. 3). Thus, tunicamycin dose up to 1 µg/mL is appropriate to study the AIS changes in live cortical neurons in our culture model.

### ER Stress Inducer Tunicamycin Causes Dose-dependent, Reversible AIS Shortening

**Fig. 4:**
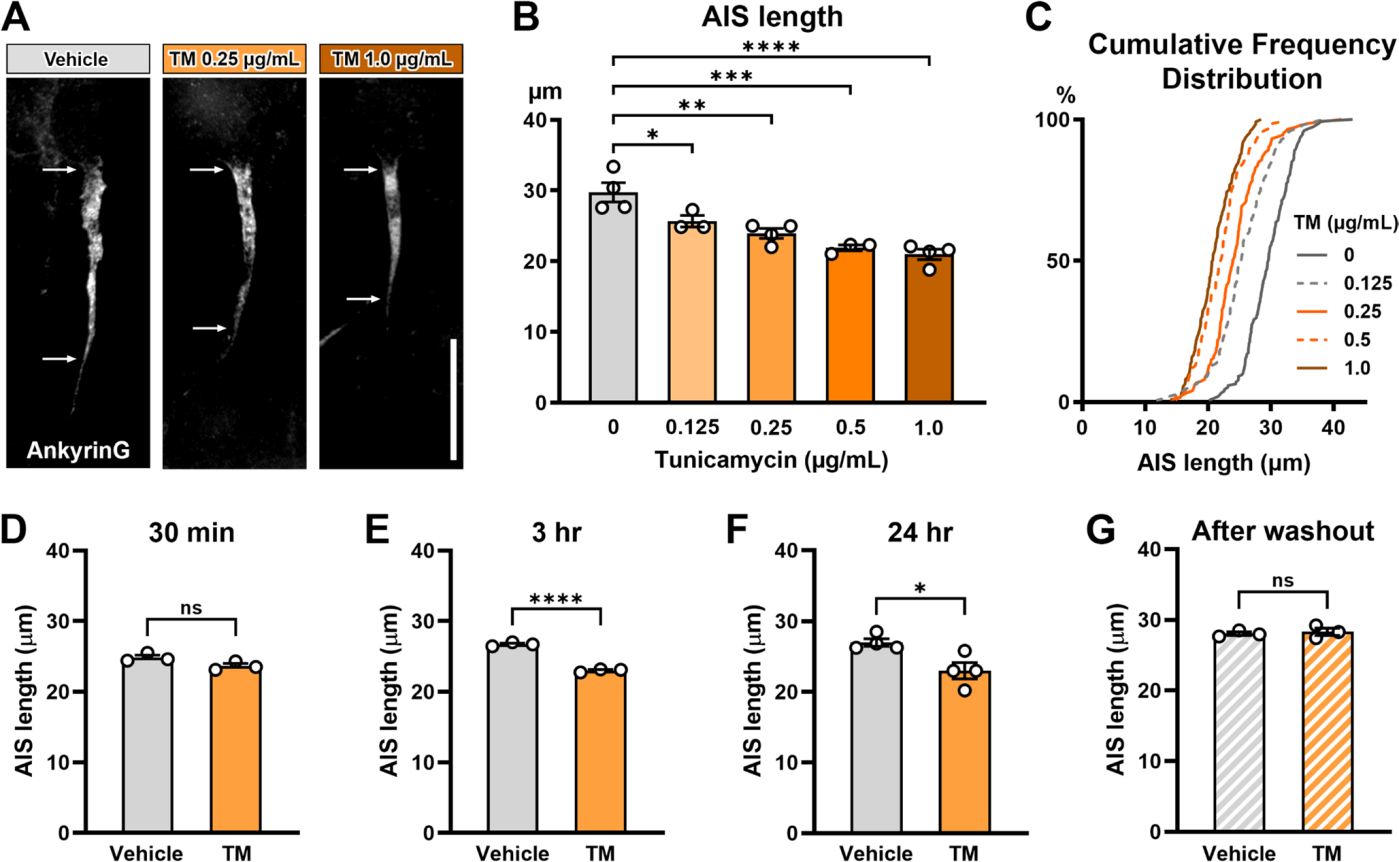
Tunicamycin induces dose-dependent, reversible AIS shortening. (**A**) Representative immunofluorescence images for the AIS protein AnkyrinG. Primary mouse E18 cortical cultures were exposed to vehicle or tunicamycin (TM) (0.25–1.0 µg/mL) for 24 h. Arrows indicate start and end positions of the AIS. Scale bar = 20 μm. (**B**) AIS length measured by AnkyrinG staining. Cells were treated with tunicamycin (0–1.0 µg/mL) for 24 h. *n* = 3–4 culture preparations; One-way ANOVA with post-hoc Dunnett’s multiple comparisons test. *p < 0.05, **p < 0.01, ***p < 0.001, ****p < 0.0001. (**C**) Cumulative frequency distribution of length of all AIS (489 AISs in TM 0 µg/mL, 379 AISs in TM 0.125 µg/mL, 443 AISs in TM 0.25 µg/mL, 337 AISs in TM 0.5 µg/mL, 465 AISs in TM 1.0 µg/mL). (**D**) AIS length after 30 min of exposure to vehicle (DMSO; 0.025%) or TM (0.25 µg/mL) (*n* = 3 culture preparations; unpaired t-test, *p* = 0.0721). (**E**) AIS length after 3-hour exposure to vehicle (DMSO; 0.025%) or TM (0.25 µg/mL) (*n* = 3 culture preparations; unpaired t-test, *p* < 0.0001). ****p < 0.0001. (**F**) AIS length after 24-hour exposure to vehicle (DMSO; 0.025%) or TM (0.25 µg/mL) (*n* = 4 culture preparations; unpaired t-test). *p < 0.05. (**G**) AIS length after replacement of vehicle (DMSO; 0.025%) or 0.25 µg/mL TM with conditioned media (*n* = 3 culture preparations; unpaired t-test; *p* = 0. 6756)

To test if ER stress induces AIS shortening independent of methylglyoxal, we exposed mouse cortical culture at DIV10 to varying doses of tunicamycin (0, 0.125, 0.25, 0.5, or 1.0 µg/mL) separately for 24 h. AIS length after tunicamycin exposure demonstrates a dose-dependent shortening of the AIS length (One-way ANOVA; *F* (4,13) = 15.05; *p* < 0.0001; post-hoc Dunnett’s multiple comparison test; *n* = 3–4 culture preparations). Compared to the cultures exposed to 0 µg/mL of tunicamycin (29.76 ± 1.368 μm), AIS length was significantly shortened with tunicamycin concentrations of 0.125 µg/mL (25.66 ± 0.8100 μm; *p* = 0.0305), 0.25 µg/mL (23.95 ± 0.7020 μm; *p* = 0.0015), 0.5 µg/mL (21.90 ± 0.4268 μm; *p* = 0.0002), or 1.0 µg/mL (20.99 ± 0.7446 μm; *p* < 0.0001) (Fig. 4A-C). The 19.5% decrease in AIS length after 0.25 µg/mL tunicamycin exposure is similar to 16% AIS shortening in prefrontal cortex in *db/db* mice (Yermakov et al. 2018) and 15.2% AIS shortening induced by 100 µM methylglyoxal in mouse cortical cultures (Fig. 2C). Therefore, we selected the tunicamycin dose of 0.25 µg/mL for subsequent experiments.

Next, we investigated the time course of tunicamycin-induced AIS shortening. After 30 min of exposure, the AIS length was comparable between tunicamycin-treated (0.25 µg/mL) cultures (23.66 ± 0.3534 μm) and vehicle-treated (DMSO; 0.025%) cultures (24.85 ± 0.3395 μm, *p* = 0.0721, unpaired t-test) (Fig. 4D). In contrast to the time course of methylglyoxal exposure (Griggs et al. 2021), there was significant shortening of the AIS after 3 h of tunicamycin treatment (23.02 ± 0.1305 μm), compared to vehicle control (26.73 ± 0.1618 μm; *p* < 0.0001, unpaired t-test) (Fig. 4E).

Finally, since low doses of tunicamycin do not cause cell death in the mouse cortical culture (Fig. 3), we assessed if tunicamycin-induced AIS shortening is reversible. After an initial 24 h of exposure, there was a significant shortening of AIS length in the cultures exposed to tunicamycin (0.25 µg/mL) (22.98 ± 1.145 μm) when compared to vehicle (DMSO; 0.025%) (26.95 ± 0.5334 μm) (*p* = 0.0199, unpaired t-test) (Fig. 4F). Following the 24-hour initial exposure to tunicamycin or vehicle, all drug media were removed from the cultures and replaced with fresh growth media. After 24 h in drug free media, AIS length in the culture initially exposed to tunicamycin (28.30 ± 0.5169 μm) was similar to that in the culture initially exposed to vehicle (DMSO; 0.025%) (28.04 ± 0.2564 μm) (*p* = 0.6756, unpaired t-test) (Fig. 4G). These data are consistent with the reversible AIS shortening induced by methylglyoxal (Griggs et al. 2021).

### ER Stress Inducer, Tunicamycin, Induces AIS Shortening in Both Excitatory and Putative Inhibitory Neurons

**Fig. 5:**
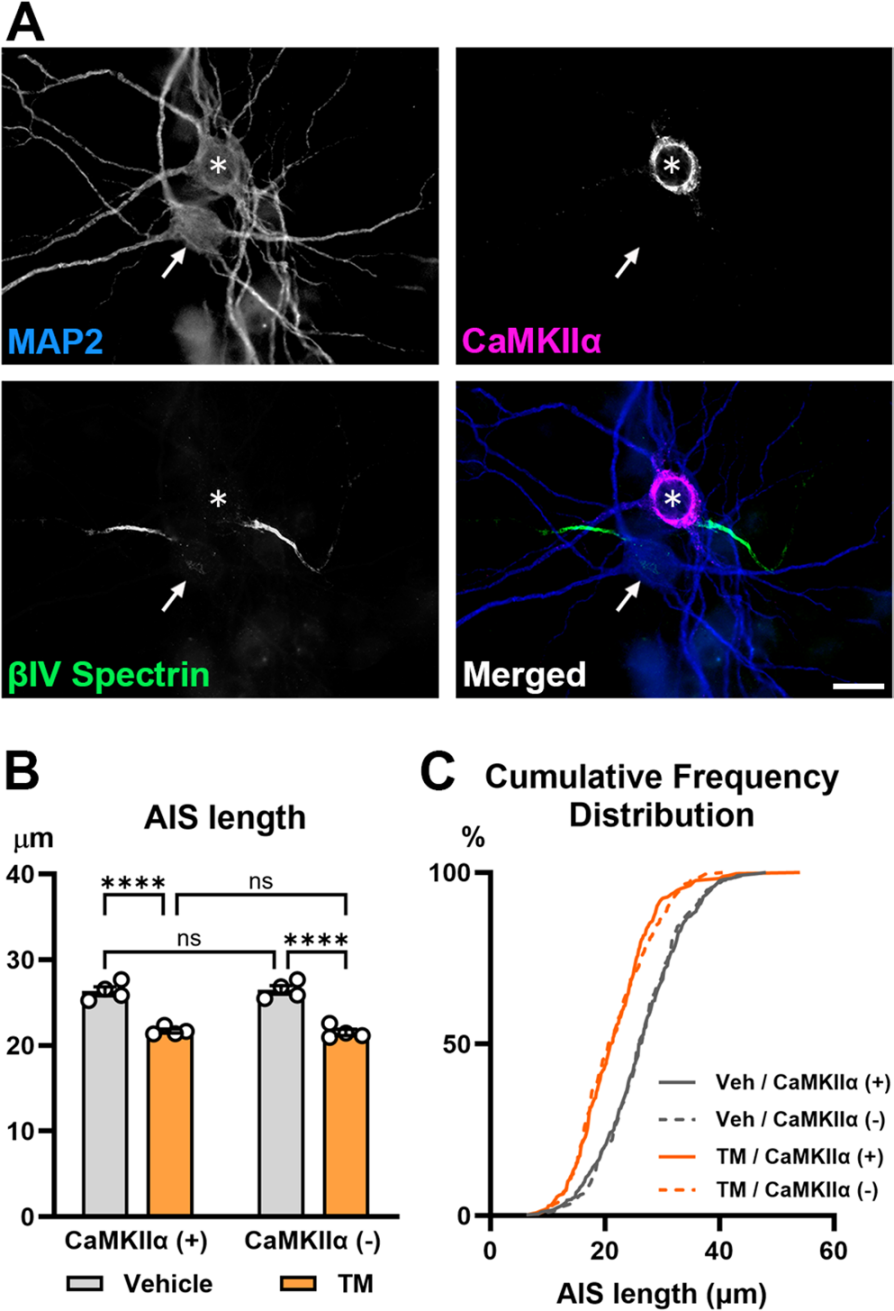
Effects of tunicamycin on AIS length in excitatory and putative inhibitory neurons. (**A**) Representative immunofluorescent images of βIV spectrin (green), CaMKIIα (violet), and MAP2 (blue) staining. E18 mouse cortical cultures were exposed to tunicamycin (0.25 µg/mL) for 24 h. CaMKIIα was used to stain excitatory neurons (asterisk), and CaMKIIα negative neurons are putative inhibitory (arrow). Scale bar = 20 μm. (**B**) AIS length measured by βIV spectrin staining in excitatory (CaMKIIα positive) and putative inhibitory (CaMKIIα negative) neurons after 24-hour vehicle (DMSO; 0.025%) or tunicamycin (TM) (0.25 µg/mL) exposure. Two-way ANOVA with post-hoc Tukey’s multiple comparison test (*n* = 4 culture preparations): Excitatory: vehicle vs. TM (*p* < 0.0001), Putative inhibitory: vehicle vs. TM (*p* < 0.0001), Vehicle: excitatory vs. putative inhibitory (*p* = 0.9952), TM: excitatory vs. putative inhibitory (*p* = 0.9941). ****p < 0.0001. (**C**) Cumulative frequency distribution of AIS lengths in excitatory (+: CaMKIIα positive) and putative inhibitory (-: CaMKIIα negative) neurons treated with vehicle (Veh) or TM (0.25 µg/mL) for 24 h (354 AISs in vehicle/CaMKIIα positive, 349 AISs in TM/CaMKIIα positive, 216 AISs in vehicle/CaMKIIα negative, 205 AISs in TM/CaMKIIα negative)

The cumulative frequency distribution of AIS length showed a leftward shift after tunicamycin treatment (Fig. 4C). This suggests tunicamycin induces generalized AIS shortening across the populations of neurons. To determine the cell-type specific effects of tunicamycin on AIS structures, we immunostained for CaMKIIα (Fig. 5A) to identify excitatory neurons (CaMKIIα positive) and putative inhibitory neurons (CaMKIIα negative). As seen with methylglyoxal (Griggs et al. 2021), tunicamycin exposure did not alter the percentage of CaMKIIα positive neurons (80.4 ± 0.483%) when compared to vehicle control (78.2 ± 1.52%) (*n* = 3 culture preparations; unpaired t-test, *p* = 0.2525). We previously showed that methylglyoxal induces AIS shortening in both of these populations of neurons (Griggs et al. 2021). Similarly, tunicamycin induced significant AIS shortening in excitatory neurons (21.68 ± 0.231 μm) when compared to vehicle control (DMSO; 0.025%) (26.34 ± 0.526 μm; *p* < 0.0001) **(**Fig. 5B, C**)**. Additionally, AIS of putative inhibitory neurons exposed to tunicamycin (21.53 ± 0.366 μm) was significantly shorter than vehicle controls (26.48 ± 0.495 μm; *p* < 0.0001). These data suggest that ER stress is sufficient to shorten the AIS in both excitatory and putative inhibitory neurons.

### Tunicamycin Does Not Alter AIS Start Location

**Fig. 6:**
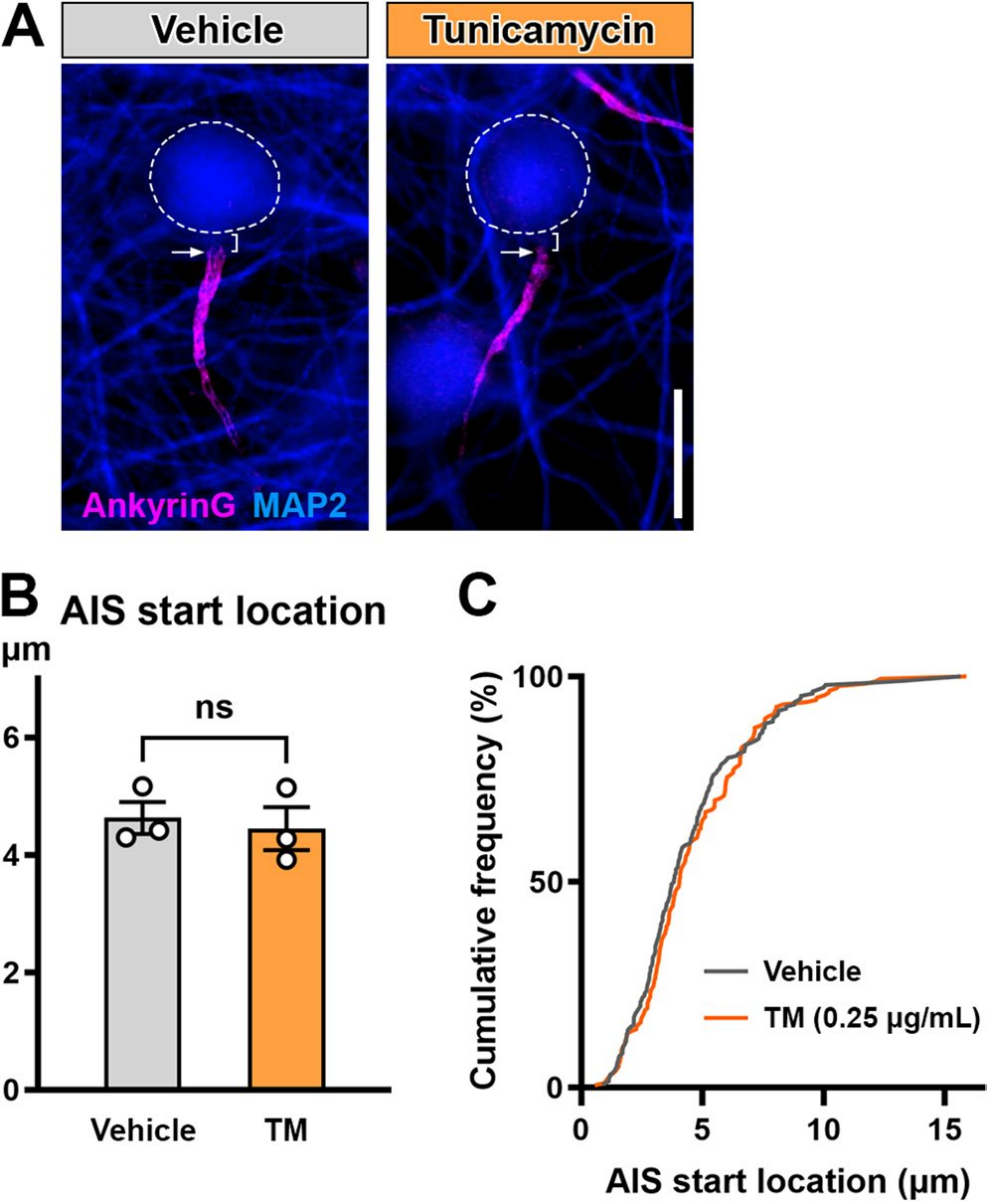
Tunicamycin does not alter the AIS start location. (**A**) Representative immunofluorescence images for the AIS protein AnkyrinG (violet) and MAP2 (blue). E18 Mouse cortical cultures were exposed to vehicle (0.025% DMSO) or tunicamycin (0.25 µg/mL) for 24 h. The soma boundary was determined by overlaying the MAP2 staining with a smooth curve along the edge of the neuron (dashed lines). Arrows indicate the start position of the AIS. Brackets indicate the AIS start location (distance between soma boundary and the start position of the AIS). Scale bar = 20 μm. (**B**) AIS start location after exposure to vehicle (0.025% DMSO) or tunicamycin (TM) (0.25 µg/mL) for 24 h (*n* = 3 culture preparations; unpaired t-test, *p* = 0.7084). (**C**) Cumulative frequency distribution of distance between soma and start position of all AIS (192 AISs in vehicle, 176 AISs in TM)

The location of the AIS can also fine-tune the excitability of neurons (Grubb and Burrone 2010; Jamann et al. 2018). Relocation of the AIS start position has been reported in disease conditions such as epilepsy (Harty et al. 2013), demyelination (Hamada and Kole 2015), inflammatory pain (Caspi et al. 2023), and Alzheimer’s disease (Hatch et al. 2017; Antón-Fernández et al. 2022; Ma et al. 2023). Therefore, we tested if ER stress can cause relocation of the AIS. The distance from soma to AIS start position was comparable between control (4.626 ± 0.2714 μm) and 0.25 µg/mL tunicamycin (4.444 ± 0.3647 μm) (*n* = 3 culture preparations; unpaired t-test, *p* = 0.7084) (Fig. 6). These data indicate that ER stress inducer tunicamycin causes AIS shortening without relocation. Consistent with these data, the AIS location was unchanged in prefrontal cortex in *db/db* mice (Yermakov et al. 2018) and in cultured mouse cortical neurons exposed to methylglyoxal (Griggs et al. 2021). The pattern of AIS geometry changes induced by ER stress is similar to the ones seen in diabetic conditions.

### PERK Inhibition Prevents Tunicamycin-Induced AIS Shortening

**Fig. 7:**
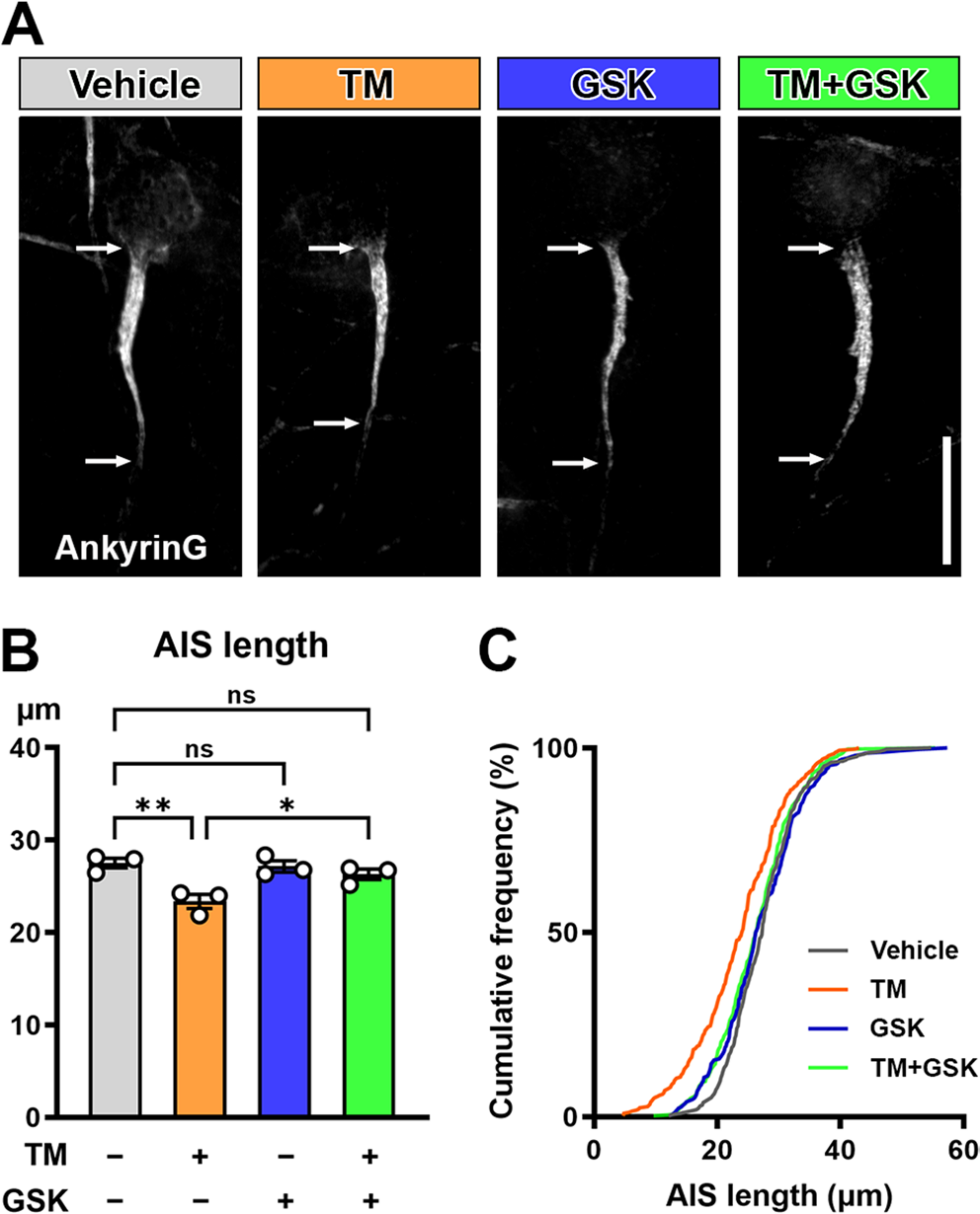
A PERK-specific inhibitor prevents tunicamycin-induced AIS shortening. (**A**) Representative images of primary mouse E18 cortical culture exposed to vehicle (0.025% DMSO), tunicamycin (TM) (0.25 μg/mL), a PERK-specific inhibitor GSK2606414 (GSK) (0.06 μM), or TM and GSK for 24 h. The cultures were immunostained for AnkyrinG. Scale bar = 20 µm. (**B**) AIS length in cultured mouse cortical neurons exposed to vehicle, TM, GSK, or TM and GSK for 24 h. Length of AIS were measured by AnkyrinG immunostaining, and average AIS length was calculated for each culture preparation (86-120 AISs per culture). Two-way ANOVA with post-hoc Tukey’s multiple comparison test (n = 3 culture preparations) yielded following p values: vehicle vs. TM (p = 0.0063), vehicle vs. GSK (*p* = 0.9745), vehicle vs. TM + GSK (*p* =0.5300), TM vs. GSK (*p* = 0.0107), TM vs. TM + GSK (*p* = 0.0409), and GSK vs. TM + GSK (*p* = 0.7585). *p < 0.05, **p < 0.01. (**C**) Cumulative frequency distribution of all AIS (308 AISs in vehicle, 325 AISs in TM, 298 AISs in GSK, 300 AISs in TM + GSK).

Three UPR pathways can be specifically targeted and have different downstream signaling effects (Ghemrawi and Khair 2020). Therefore, uncovering the pathway responsible for AIS shortening could allow for development of more targeted therapeutics. Among three UPR branches, the PERK/eIF2α axis is most well studied as a potential therapeutic target to mitigate symptoms in neurodegenerative conditions such as Alzheimer’s disease (Halliday et al. 2017; Ohno 2018; Rozpędek-Kamińska et al. 2020; Smedley et al. 2021). For example, inhibition of PERK activity by a PERK-specific inhibitor GSK2606414 in the hippocampal CA1 region enhances memories in middle-aged mice (Sharma et al. 2018). Importantly, there was an inverse correlation between AIS length and P-PERK/PERK ratio (Fig. 2G). To test if the PERK pathway mediates AIS shortening, we exposed mouse cortical cultures to either vehicle (DMSO 0.025%), tunicamycin (0.25 µg/mL), GSK2606414 (0.06 µM), or tunicamycin and GSK2606414 for 24 h and examined the AIS morphology (Fig. 7A). There was a statistically significant interaction between the effects of tunicamycin and GSK2606414 on AIS length (two-way ANOVA; *F* (1,8) = 7.066; *p* = 0.0289; post-hoc Tukey’s multiple comparison test; *n* = 3 culture preparations) (Fig. 7B). The AISs of cultures exposed to tunicamycin (23.37 ± 0.7546 μm) were significantly shorter than vehicle control (27.52 ± 0.5219 μm) (*p* = 0.0063). The AISs in tunicamycin-treated cultures were also shorter than tunicamycin and GSK2606414 co-treated cultures (26.29 ± 0.5682 μm) (*p* = 0.0409). No difference in AIS length was found between tunicamycin and GSK2606414 co-treated cultures and vehicle controls (*p* = 0.5300). These data suggest that the PERK pathway is the key mediator for ER stress-induced AIS shortening.

## Discussion

The AIS regulates neuronal output as the site where action potentials are initiated. Recent studies indicate that subtle changes in AIS geometry (e.g. shortening of the AIS length) can significantly affect the neuronal excitability and firing properties (Jamann et al. 2018). Given the importance of the AIS in regulating action potential initiation, it is not surprising that structural alterations in the AIS play important roles in various neurodegenerative conditions (Buffington and Rasband 2011; Huang and Rasband 2018; Garrido 2023; Fréal and Hoogenraad 2025). We have previously shown that the AIS is shortened in the prefrontal cortex and hippocampus in the T2DM model *db/db* mice when these mice show impairment of cognitive flexibility (Yermakov et al. 2018, 2019). Since AIS shortening leads to decrease of neuronal excitability (Baalman et al. 2013; Evans et al. 2015; Jamann et al. 2021), it is conceivable that AIS shortening contributes to the pathophysiology of brain complications in T2DM. However, the cellular and molecular mechanisms of AIS disruption in disease conditions remain poorly understood. Here, we demonstrate that ER stress induces shortening of AIS length. This conclusion is supported by three experimental results in mouse cortical culture. First, the diabetes factor methylglyoxal elevated ER stress markers GRP78 and P-PERK/PERK ratio. Second, ER stress inhibition with 4PBA prevented methylglyoxal-evoked AIS shortening. Finally, ER stress induction with tunicamycin caused dose-dependent AIS shortening, an effect that was prevented by the PERK inhibitor GSK2606414. Our findings will contribute to a better understanding of the pathophysiology of brain complications due to T2DM and potentially other neurodegenerative conditions that impact AIS geometry.

### ER Stress is Required for Methylglyoxal-Induced AIS Shortening

Methylglyoxal, a reactive α-dicarbonyl compound generated by glycolysis, has been implicated in neurodegenerative conditions such as diabetic brain complications and Alzheimer’s disease (Allaman et al. 2015; Yang et al. 2022). We have identified methylglyoxal as a potential mediator of AIS shortening in diabetic conditions (Griggs et al. 2021). Here we show that methylglyoxal induces an increase in GRP78 protein levels in primary mouse cortical cultures (Fig. 1), at the same dose (100 µM) required for AIS shortening (Fig. 2B-D) (Griggs et al. 2021). Because GRP78 is upregulated in ER stress conditions, this suggests that methylglyoxal-induced AIS shortening occurs concomitantly with ER stress and UPR activation. This is further supported by our finding that methylglyoxal increases the ratio of activated PERK to inactivated PERK in our culture model (Fig. 2E, F). Upon activation, PERK is autophosphorylated and P-PERK then initiates downstream signaling to attenuate global protein synthesis and decrease ER protein load (Rozpędek-Kamińska et al. 2020). Methylglyoxal increases the proportion of PERK that has been autophosphorylated, suggesting presence of ER stress and UPR in conditions of methylglyoxal-induced AIS shortening. Importantly, co-exposure to an established ER stress inhibitor 4PBA prevented the AIS shortening as well as increase of P-PERK/PERK ratio by methylglyoxal (Fig. 2). This suggests that ER stress and UPR activation is required for the AIS shortening in diabetic conditions. 4PBA is widely used to inhibit ER stress because of its ability to function as a chemical chaperone. Similar to the action of the endogenous protein chaperone GRP78, 4PBA binds to aberrantly exposed hydrophobic regions in misfolded proteins to prevent aggregation within the ER (Kolb et al. 2015). Similar to our results, in cultured retinal pigment epithelial cell line, inhibition of ER stress by 4PBA reduced the methylglyoxal-induced intracellular events and cell death (Chan et al. 2016). Furthermore, in vivo administration of 4PBA attenuated cognitive deficits in T2DM rats by high-fat diet and a single small dose of streptozotocin (Wang et al. 2016), and in a streptozotocin-induced diabetic mouse model (Kong et al. 2018). ER stress occurs in the T2DM model *db/db* mice (Sims-Robinson et al. 2012; Li et al. 2018, 2020; Ye et al. 2018; Hu et al. 2020; Wu et al. 2020), in association with AIS shortening and impairment of cognitive flexibility (Yermakov et al. 2018, 2019). Collectively, these findings support the idea that ER stress is a key cellular event that mediates AIS shortening, presumably contributing to development of cognitive impairment in T2DM.

### ER Stress is Sufficient for Induction of AIS Shortening

We found that the established ER stress inducer tunicamycin caused dose-dependent AIS shortening in mouse cortical cultures (Fig. 4A-C). ER stress and the UPR activation can trigger apoptosis (Ghemrawi and Khair 2020). However, neuronal apoptosis was not driving AIS shortening, because low-dose tunicamycin (1.0 µg/mL or less) did not affect cellular viability in our neuronal cultures (Fig. 3), and the tunicamycin-induced AIS shortening was recoverable (Fig. 4F, G). The time course of AIS shortening by tunicamycin differs from methylglyoxal induced AIS shortening. There is no change in AIS length after 30 min–3 h of methylglyoxal exposure (Griggs et al. 2021). Conversely, tunicamycin induces AIS shortening more rapidly, with significant shortening after 3 h of exposure (Fig. 4E). The reason for this discrepancy is not understood.

Our data suggest that tunicamycin causes generalized AIS shortening across the entire neuronal populations including excitatory and putative inhibitory neurons (Figs. 4C and 5). These features of tunicamycin-induced AIS shortening are similar to that induced by methylglyoxal (Griggs et al. 2021). Furthermore, we did not see changes in AIS location, another AIS structural factor affecting neuronal excitability, in the mouse cortical cultures exposed to tunicamycin (Fig. 6) or methylglyoxal (Griggs et al. 2021), or in the *db/db* mice (Yermakov et al. 2018). These findings indicate that ER stress is sufficient to induce AIS structural changes similar to those in the diabetic conditions (Yermakov et al. 2018, 2019; Griggs et al. 2021). Importantly, AIS shortening caused by sub-lethal levels of methylglyoxal (Griggs et al. 2021) or tunicamycin was reversible after washout of these drugs (Fig. 4F, G). Apoptotic cell death was not observed in *db/db* mouse brains when these mice show cognitive impairment and AIS shortening (Yermakov et al. 2018). Therefore, the AIS shortening in diabetic brains could be reversible by treatment targeting ER stress, suggesting its potential for early intervention.

### PERK Pathway in UPR Mediates ER Stress-Induced AIS Shortening

Among the three UPR branches, studies investigating the mechanisms for neurodegenerative conditions have focused on the PERK pathway (Halliday et al. 2017; Ohno 2018; Rozpędek-Kamińska et al. 2020; Smedley et al. 2021). Previous studies showed that methylglyoxal activates PERK pathway in vitro (Palsamy et al. 2014; Barragán-Iglesias et al. 2019; Xue et al. 2024). Methylglyoxal-induced AIS shortening as well as PERK activation are prevented by the ER stress inhibitor 4PBA (Fig. 2). Tunicamycin activates all three UPR pathways including PERK in vitro (Lu et al. 2016; Qureshi et al. 2023). Co-incubation of the mouse cortical culture with tunicamycin and the PERK-specific inhibitor GSK2606414 prevented AIS shortening (Fig. 7), suggesting that the PERK pathway mediates the ER stress-induced AIS shortening. However, the molecular mechanisms through which activation of the PERK pathway leads to AIS shortening remain to be elucidated. P-PERK phosphorylates α subunit of eukaryotic translation initiation factor 2 (eIF2α) to decrease ER protein load by inhibiting protein synthesis (Halliday et al. 2017; Ohno 2018; Rozpędek-Kamińska et al. 2020; Smedley et al. 2021). Phosphorylated eIF2α also upregulates activating transcription factor 4 (ATF4), which in turn promotes transcription of target genes in diverse cellular processes, including protein folding, amino acid metabolism, or autophagy. ER stress is known to trigger a variety of cellular responses without inducing cell death. For example, sub-lethal doses of tunicamycin induce ATF4-dependent autophagic degradation of stromal interaction molecule 2, a protein implicated in dendritic spine formation, and reduce dendrite arbor in primary rat neuronal cultures (Zhou et al. 2019). Future studies are needed to determine the mechanisms of how sub-lethal ER stress leads to AIS shortening and how these structural changes affect neuronal circuit function. Additionally, it remains to be determined whether PERK inhibition can ameliorate methylglyoxal-evoked AIS shortening in vitro, as well as the AIS shortening observed in diabetic animal models in vivo. Previous studies show PERK activation in *db/db* mouse brains (Li et al. 2018; Ye et al. 2018; Hu et al. 2020; Wu et al. 2020). Indeed, treatment with GSK2656157, a specific inhibitor of the PERK pathway, improved learning and memory function in diabetic *db/db* mice (Wu et al. 2020). These previous studies focused on neuroinflammation, synaptic alterations, neurodegeneration, and neuronal apoptosis as potential mechanisms for ER stress-induced cognitive dysfunction (Li et al. 2018; Ye et al. 2018; Hu et al. 2020; Wu et al. 2020). In addition to these pathological changes, our results highlight the importance of investigating AIS as the neuronal structure influenced by ER stress in diabetic conditions.

### Limitations of the Study and Unanswered Questions

Although our data provided key insights into the mechanism of AIS shortening in diabetes, we must acknowledge the limitations of this study and unanswered questions to address in future investigations. For instance, acute ER stress induction in cortical cultures may not be generalizable to the chronic ER stress response seen in a more complicated in vivo diabetic brain condition. Similarly, there are supporting cells such as microglia that play critical roles in vivo (Baalman et al. 2015; Benusa and Lafrenaye 2020) but are not represented in our culture model. A recent study indicated the roles of alterations in astrocytes in mediating AIS structural changes (Benitez et al. 2024). Future research investigating the effects of ER stress and PERK inhibition using in vivo models, where endogenous glial cells are present, will be necessary to further confirm the impact of ER stress and PERK activation on AIS shortening. Finally, because the AIS has a critical role in neuronal homeostatic plasticity, functional deficits could be evaluated alongside AIS geometry changes in response to ER stress.

### Conclusions

Our results indicate that ER stress, potentially through the PERK UPR pathway, is the key mechanism mediating AIS shortening in cultured mouse cortical neurons. In addition to T2DM, AIS shortening is associated with many neurodegenerative conditions including Alzheimer’s disease (Marin et al. 2016; Best et al. 2023; Ma et al. 2023; Benitez et al. 2024), aging (Atapour and Rosa 2017; Ding et al. 2018), traumatic brain injury (Baalman et al. 2013; Vascak et al. 2017), neuropathic pain (Shiers et al. 2018), attention-deficit hyperactivity disorder (Usui et al. 2022), and autism-spectrum disorder (Usui et al. 2022). These observations indicate that altered AIS geometry (e.g., AIS shortening) is a common mechanism of brain dysfunction in various neurodegenerative conditions. Importantly, ER stress has been shown to be involved in the pathogenesis of Alzheimer’s disease (Ajoolabady et al. 2022), aging (Martínez et al. 2017), traumatic brain injury (Yang et al. 2024), neuropathic pain (Kawanaka et al. 2024), and autism-spectrum disorder (Kawada and Mimori 2018). These insights into the AIS alterations and ER stress will provide fundamental knowledge of the pathophysiology of cognitive impairment in T2DM and potentially in other neurodegenerative conditions, ultimately inspiring future therapeutic studies for patients living with these conditions.

## Supplementary Information

Below is the link to the electronic supplementary material.

Supplementary File 1 (PDF 136 KB)

## Data Availability

The datasets analyzed in this study are available from the corresponding author upon reasonable request.
