# Supplementary material for: Endoplasmic Reticulum Stress Mediates Axon Initial Segment Shortening: Implications for Diabetic Brain Complications
Source: J Mol Neurosci. 2025 Dec 19;76(1):1. doi: 10.1007/s12031-025-02448-y (PMC12717103; doi:10.1007/s12031-025-02448-y)

Supplementary Information

Shelby et al.

Journal of Molecular Neuroscience

“Endoplasmic reticulum stress mediates axon initial segment shortening: implications for diabetic brain complications”

Supplementary Figure S1. Original immunoblot for Fig. 1A

Original full-size immunoblot of P0 mouse cortical culture lysates stained for GRP78.

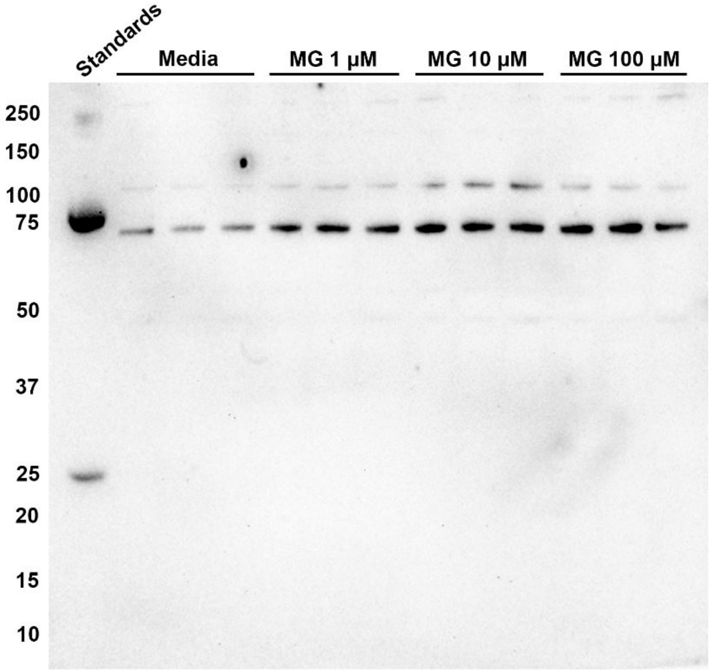

Supplement: Supplementary file 1 — (PDF 136 KB) [file 12031_2025_2448_MOESM1_ESM.pdf]
